# Laclau and Mouffe's Discourse Theory: Professionalism as an Empty Signifier for Nursing

**DOI:** 10.1111/nup.70034

**Published:** 2025-08-12

**Authors:** Sarah M. Ramsey

**Affiliations:** ^1^ Birkbeck, University of London London UK; ^2^ School of Health Sciences The University of Manchester Manchester UK

**Keywords:** discourse, empty signifier, hegemony, Laclau, Mouffe, professionalism

## Abstract

Chantal Mouffe and Ernesto Laclau made significant developments in discourse theory, based on the premise that *all* practices and objects are discursive, deriving meaning through existence in a wider context. This paper introduces the work of Laclau and Mouffe, explicating the main tenets of discourse theory through an example chosen from the nursing discourse, ‘professionalism’, and exploring how this serves as an empty signifier. The construction of a discourse is explored, from the building blocks of individual signs and moments to the totality established when each sign is fixed in relation to other signs and all other possible meanings excluded. The empty signifier represents a lack or absence, stabilising the discourse in a move towards hegemony. The question is posed as to whether, as an empty signifier, professionalism has resulted in a successful hegemonic formulation. Nursing has always struggled to be ‘fully constituted’ with an antagonistic frontier delineating ‘women's work’ of caring and nurturance from the cost‐effectiveness of evidence‐based practice. However, the lack of a coherent identity for nursing is highlighted as the empty signifier defines us merely as ‘not unprofessional’. Identification of the empty signifier ‘professionalism’ and exploration of the ensuing hegemonic formation of ‘professional’ nursing identity demonstrates that the work of Laclau and Mouffe has significant relevance to nursing. Exploration of further areas of the nursing discourse through the lens of discourse theory is encouraged.

## 1 Introduction

Chantal Mouffe (1943–) and Ernesto Laclau (1935–2014) met in 1973 at the University of Essex; based on their shared interests in socialism and historical affiliation with political struggles, they established a school of post‐structural discourse theory. Laclau and Mouffe's work considers timeless concepts such as the use of language, societal structure and influence of ideology, with themes of identity, inclusivity and challenge of power relations which are highly relevant to nursing today. In 1985, Mouffe and Laclau published *Hegemony and Socialist Strategy: Towards a Radical Democratic Politics*, a starting point for their discourse‐theoretical approach to politics (Smith 1998). In this paper, I aim to introduce Laclau and Mouffe's theory of discourse as outlined in *Hegemony*, demonstrating the applicability of this to nursing through consideration of the term ‘professionalism’ as an empty signifier within the nursing discourse.

I will explore the previous (limited) use of discourse theory within the nursing literature and provide some background to the development of Laclau and Mouffe's work. An overview of discourse theory is provided, following which the discursive articulation of nursing identity is considered, centred around the exploration of the concept of ‘professionalism’ as an empty signifier. This serves to explicate the relevance of Laclau and Mouffe's theory to nursing discourse and involves a deeper exploration of the main concepts of discourse theory and how they can be applied in this instance. This includes the articulation of discourse through moments and nodal points, the logic of difference and equivalence, the concept of the empty signifier, antagonistic frontiers and the role of hegemony. To conclude the paper, the use of Laclau and Mouffe's discourse theory as a philosophy for nursing is considered.

## 2 Laclau and Mouffe in the Nursing Literature

There has been limited engagement with the work of Laclau and Mouffe in the nursing literature. Cloyes (2002, 204) considered care ethics and political theory of care, describing care as ‘the articulated foundation of what [nurses] do … constructed by linking contingent elements into what functions as a naturalized and coherent discourse’. Österlind et al. (2011) used discourse theory to explore carer thoughts and feelings about death in the nursing home setting. Oute (2018) drew on discourse theory to consider the situation of psychiatric nursing discourse within ideological structures of science, ethics and gender. In an exploration of the use of material feminist theory in nursing, Aranda (2019) suggested that radical democracy could provide a way to incorporate both matter and meaning while justifying a greater level of practitioner involvement in addressing inequalities in health care. Traynor (2019) considered nursing work from a Marxist perspective, critiquing the concepts of caring and autonomy in terms of ideology and antagonism; in 2022, his Freudian critique of Nursing work made reference to the work of Althusser. Sabab et al. (2020) explored the use of language in nursing treatment plans through the lens of discourse theory. Re‐organisation of Dutch nursing work and the response of nurses to this has been explored through the work of Laclau and Mouffe to study the interplay between professional grievances, emergence of group identities and organisation of collective dissent (Felder et al. 2022).

## 3 An Introduction to Discourse Theory

In the United Kingdom in 1985, the trade union movement, along with leftist political parties, was losing ground to right‐wing forces. The New Right was a political tradition that rejected the conventions of postwar politics, such as paternalistic tendencies, aiming to re‐instate individualistic values previously associated with the liberal ‘free market’ of earlier times (Williams 2021). The left struggled to respond to this clear and coherent neo‐liberalist vision. Simultaneously, activist movements such as those of feminists, environmentalists and pacifists were emerging, taking on the political struggles which the left had so far inadequately addressed. This redefined ‘the very meaning of leftist politics’ through increased emphasis on social issues such as gay rights, opposing the prevailing authoritarian structures of society in a move towards cultural liberalism and shifting focus away from social class and workers' rights (Smith 1998). Radical democracy was Laclau and Mouffe's proposed response to the ‘crisis of the left’ in Western Europe, including aspects of both socialism and liberal democracy (Laclau and Mouffe 1985; Smith 1998). Socialism is based on the concept that human nature is malleable and all people are potentially equal, their character a product of circumstance. In opposition to capitalism, socialism advocates for means of production, exchange and distribution to be regulated or owned by society as a whole (Roberts and Sutch 2012). Liberal democracy combines a democratic political system of elected representatives with liberalist principles of individual rights, free exchange and competition between individuals and limits to the power of the state over the public sphere (Bass 2005). Laclau and Mouffe viewed radical democracy as an attempt to grasp the ‘roots’ of democracy, which had previously been obscured, including equal participation of ‘the people’ in power. They felt that other forms of democracy were oppressive and expanded liberal notions of democracy based on equality and freedom to include difference. Built around dissent and difference, radical democracy aimed to highlight and challenge oppressive power relations in society (Dahlberg 2012).

Laclau and Mouffe (1985) described an impasse in Marxist theorisation in the mid‐1970s, following the rich creativity of the 1960s. Their approach has been described as post‐Marxist, being based on a deconstruction of Marxism and movement away from essentialism (the concept that everyone and everything has an innate ‘essence’ of attributes which define them, determining membership of a specific group [Phillips 2010]) through the concepts of hegemony and antagonism (Biglieri and Perelló 2011). Hegemony can be defined as dominance or leadership, particularly by a social group or state over others (Torfing 1999); Howarth (1995, 124) notes that ‘very simply, hegemony is achieved if and when one political project or force determines the rules and meanings in a particular social formation … it is about which political force will decide the dominant forms of conduct and meaning in a given social context’. According to Laclau and Mouffe (1985, 3), ‘behind the concept of “hegemony” lies hidden something more than a type of political relation complementary to the basic categories of Marxist theory. In fact, it introduces a logic of the social which is incompatible with those categories’. They therefore established hegemony as central to the discursive theory later elucidated, while situating their theory as post‐Marxist. The ‘logic of the social’ proved to be fundamental to their work, as will be discussed later.

Laclau and Mouffe acknowledged that they were working in an intellectual genealogy made possible by the work of Marxist philosopher and politician Antonio Gramsci (1981–1937), a founding member of the Italian Communist Party. According to Laclau, Gramsci's most significant contribution to political theory came from his use of the concept of hegemony to reformulate socialist strategy, an attempt to ‘move in the direction of a post‐Marxism able to deal with the fragmented and incomplete character of social identities in the contemporary world’ (Laclau 1998, 468; Forgacs 2000, 192). Laclau and Mouffe (1985, 3) note that expanding on Gramsci's work provided them with ‘an anchorage from which contemporary social struggles are thinkable in their specificity’. They describe how a hegemonic movement works through the construction of discourse, running counter to antagonism to weave a variety of unfixed attitudes and demands into fixed positions, with ideology playing a vital role (Laclau and Mouffe 1985; Torfing 1999). Laclau and Mouffe's conceptualisation of discourse has roots in Louis Althusser (1918–1990) theory of ideology as integral to the structure of society, developed through confrontation with elements of Marxist theory (Althusser 1971; Howarth 1998). Like Marx, Althusser stressed the opposition between science and ideology, with ideology being a ‘system of representations’ (‘images, myths, ideas, or concepts’) (Althusser 1969, 231; Leopold 2013). These ‘representations’ of nature or society guide us in accordance with ‘assigned tasks’ of societal membership (Althusser 1990, 24–25). According to Althusser, ideology was necessary to maintain social order, with individuals formed in accordance with the social structure; members of society are ‘formed, transformed, and equipped to respond to the demands of their conditions of existence’ (Althusser 1969, 235). As Leopold (2013, 32) describes, ‘the efficacy of ideology is portrayed in terms of its success in cementing individuals to the social role that they are allocated by the particular social structures that obtain, thereby ensuring the reproduction of those social structures’ (Althusser 1990, 25).

## 4 Articulation of Discourse

Introducing their theory of discourse, Laclau and Mouffe (1985, 93) reject ‘the distinction between discursive and non‐discursive practices … every object is constituted as an object of discourse’. The concept of discourse elaborated by Laclau and Mouffe therefore extends beyond language to incorporate social phenomena; social practices are fluid and changeable, they cannot be fixed, with associated meanings changing over time. Discourse theory is based on the premise that *all* practices and objects are discursive; they derive meaning through existence in a wider context; the concept of professionalism in nursing, therefore, may be seen as evolving within and as a response to this complex wider social context.

According to Laclau (1993), in discourse theory, the possibility of thought and action depends on the pre‐existence of a structured field of meaning. The subject is no longer positioned as a source of meaning, being rather ‘just one more particular location within a meaningful totality’ (p. 433). Even ‘natural’ subject positions, which may seem to have only one possible meaning, may be subversively redefined; it is a sign of powerful hegemonic normalisation which causes them to appear natural. Discourse theory prompts us to search for hidden forces of power and institutionalisation behind apparent ‘nature’ (Smith 1998).

Laclau and Mouffe describe articulation as a

practice establishing a relation among elements such that their identity is modified as a result of the articulatory practice. The structured totality resulting from the articulatory practice, we will call discourse.

(1985, 91)

In simpler terms, articulation describes how we connect objects, words and ideas in specific ways when acting or speaking to express meaning. If these connections are continuously repeated, patterns will start to form, providing a structure for the social world (Jacobs 2018). Discourse theory provides a useful structure to explore how nursing identity may be discursively constructed through this process of articulation.

## 5 Professionalism and Nursing

To simultaneously explicate the various components of discourse theory and demonstrate the relevance of Laclau and Mouffe's theory to nursing, I will explore the discursive articulation of nursing identity, centred around the idea of ‘professionalism’ as an empty signifier. There has been much deliberation around the nature of, and necessity for, professionalism within nursing practice, with multiple definitions of the term; historians, sociologists and nurses have debated the status of nursing from both within and outside the profession (Ghadirian et al. 2014). Using discourse theory, I will therefore consider how professionalism has served as a structuring element for the construction of nursing identity, while simultaneously representing the *lack* of identity it conceals. In nursing, it has proven difficult to ascertain a clear identity associated with the subject position ‘nurse’; the concept of nursing itself has been widely debated. Hunt and Wainwright (1994) highlighted difficulties in defining the role expansion of the time due to a lack of specific attributes to characterise ‘nursing’. More recently, van der Cingel and Brouwer (2021) explored dualism within a nursing identity shaped through historical and social influences, describing nursing as a ‘social construct’ impacted by stereotyping. Much of the relevant nursing literature refers to ‘professional identity in nursing’ rather than simply ‘nursing identity’, a convention which seems to have grown in popularity over time. Professional identity includes the skills, knowledge, beliefs, values and attitudes shared by those within a professional group. Such identity evolves through a continuous process, influenced by factors such as socialisation and clinical experience (Matthews et al. 2019). Philippa et al. (2021) describe professional identity in nursing as a construct constituting personal beliefs, values and attitudes combined with understandings and characteristics of the nursing profession itself. They suggest that professional identity develops through clinical experience, understanding of the role and self‐understanding, with development influenced by professional and personal factors. To explore the role of professionalism in the construction of nursing identity, it is first necessary to consider the mechanisms through which discourse is articulated.

## 6 Moments in Discourse

Laclau and Mouffe derived discourse theory from three main currents within the philosophy of the 20th century, phenomenology, structuralism and analytical philosophy. Structuralism emerged from the work of linguist Ferdinand de Saussure (1857–1913), who rejected the referential theory of language, where the meaning of a word is that which it *refers to*, and language is used to assign names to objects and ideas. Instead of a thing and a name, Saussure used the concepts of signifier (the sound of a word) and signified (the idea of a thing), together producing a linguistic sign (Saussure 1959; Smith 1998). A sign is not a real object in itself, but rather an arbitrary component in a system of language; signs are distinct from ‘real’ objects. As Laclau describes,

language constitutes a system in which no element can be defined independently of the others. Language is form and not substance – that is, each element of the system is exclusively defined by the rules of its combinations and substitutions with the other elements.

(1993, 431)

Signs can therefore only be understood through the complex system of which they are a part (Linden 2023).

Within a discourse, each sign is classed as a ‘moment’ with meaning derived from its relationship with other signs. Jørgensen and Phillips (2011) provide a visual image of knots in a fishing net. A sign without a fixed meaning is described as an ‘element’ and the articulation of discourse attempts to create moments from elements, though this is never wholly successful (Laclau and Mouffe 1985). Discourse is established as a totality when each sign is fixed in relation to other signs and all other possible meanings are excluded (Jørgensen and Phillips 2011). Many signs could be viewed as constitutive of the nursing discourse; some ‘elements’ which derive specific meaning when situated within the nursing frame of reference, thereby becoming ‘moments’ (at least within the British nursing discourse) might include such terms as ‘special’, ‘observation’, ‘round’, ‘turn’ and so on. However, such terms retain alternative usage within other discourses; as Laclau and Mouffe (1985, 97) observe, ‘transition from the “elements” to the “moments” is never entirely fulfilled … there is no social identity fully protected from a discursive exterior that deforms it and prevents it becoming fully sutured’. To continue with the previous examples, while ‘round’ may have specific connotations within nursing, this has not prevented the use of the term within alternative discourses, such as ‘a round of golf’. Identification of such elements does, however, highlight the building blocks through which the nursing discourse, and professional nursing identity, are constructed.

## 7 Professionalism: Articulation of a Discourse

**Figure 1:**
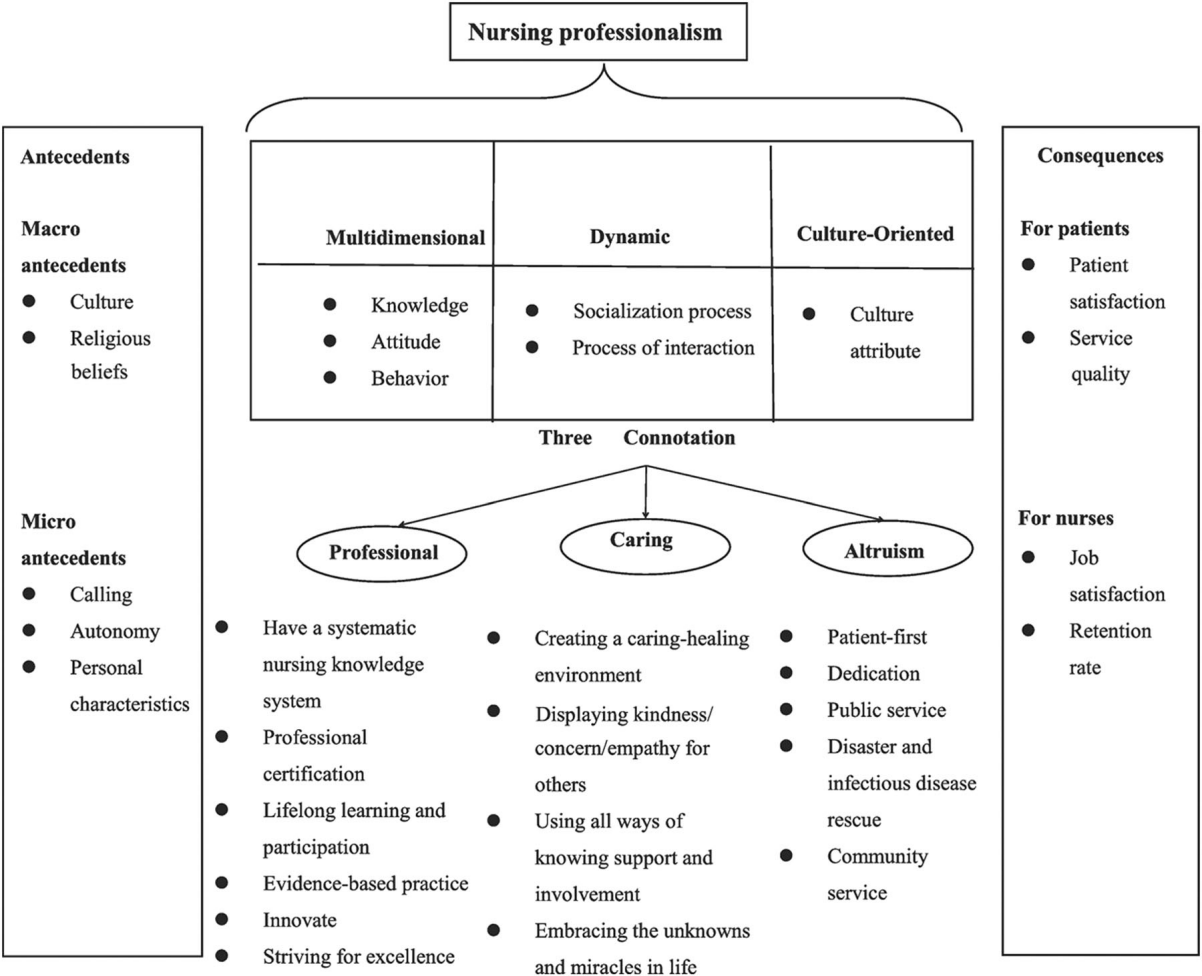
Antecedents, attributes and consequences of Nursing Professionalism. *Source:* Cao et al. (2023, 5), licensed under a Creative Commons Attribution 4.0 International License, http://creativecommons.org/licenses/by/4.0/, minor amendments to correct proofing errors.

To consider the proposed example of ‘professionalism’ within nursing, it is helpful to examine the recent concept analysis conducted by Cao et al. (2023). Their study was based on the premise that while professionalism played a vital role in clinical nursing, a clear conceptual understanding of this was lacking. They analysed the concept of nursing professionalism as defined in 138 previous studies, ending with a circular conclusion that ‘the three attributes of nursing professionalism are multidimensional, dynamic, and culture oriented … nursing professionalism is defined as providing individuals care based on the principles of professionalism, caring, and altruism’ (p. 1). Cao et al. (2023, 5) present a diagram of the antecedents, attributes and consequences of nursing professionalism, as shown in Figure 1.

Laclau and Mouffe (1985) describe how articulation of a discourse occurs through the construction of ‘nodal points’ around which meaning can be partially fixed; it is the relationship between the sign and the nodal point which provides meaning (Howarth 1998; Jørgensen and Phillips 2011). A simple example of this could be the terms ‘hospital ward’, ‘pressure relief’ or ‘nursing care’ which within the nursing discourse would act as nodal points partially fixing the meaning of the signs previously listed into ‘moments’; it is through relation to such signifiers that the element ‘turn’ takes on nursing‐specific meaning (‘turning’ patients to prevent pressure damage) to the exclusion of other meanings, for example, taking turns, wood‐turning and so on.

Introduction of nodal points transforms the image of a discursive network from a fishing net to a spider web, with peripheral signifiers connected to each other, to those which are more central, and eventually to a nodal point at the centre. Each discourse may have multiple nodal points, for example, law, equality and freedom in the discourse of liberal democracy (Jacobs 2018). While not seemingly the intention of the authors, Figure 1, Cao et al.'s (2023, 5) diagram of nursing professionalism helpfully brings to mind the image of this spider‐web of signifiers. While nodal points provide singular points of anchoring, wider articulation of a discourse is explained through the concept of ‘chains of equivalence’; signifiers derive meaning through becoming part of such chains. Smith (1998, 88) explains that ‘wherever different subject positions are symbolically located together in opposition to another camp, such that their meanings are subsequently transformed by their overlapping identifications with partially shared sets of beliefs, then we are dealing with an articulated chain of equivalence’. Establishing a signifier within an equivalential chain increases the extent to which it is ‘fixed’ and reduces the opportunity for other possibility of meaning, as emphasis is placed on the specific meaning required to establish the equivalence (Torfing 1999). For example, a nodal point of ‘nursing values’ creates a chain of equivalence between elements such as compassion, care and kindness. In the study conducted by Sabab et al. (2020) regarding care planning, the discourse was problem‐focused and ‘problem’ was identified as a nodal point; only information relating to complications of treatment was recorded; no record was made if no problems occurred, leading to omissions within the patient record.

**Figure 2:**
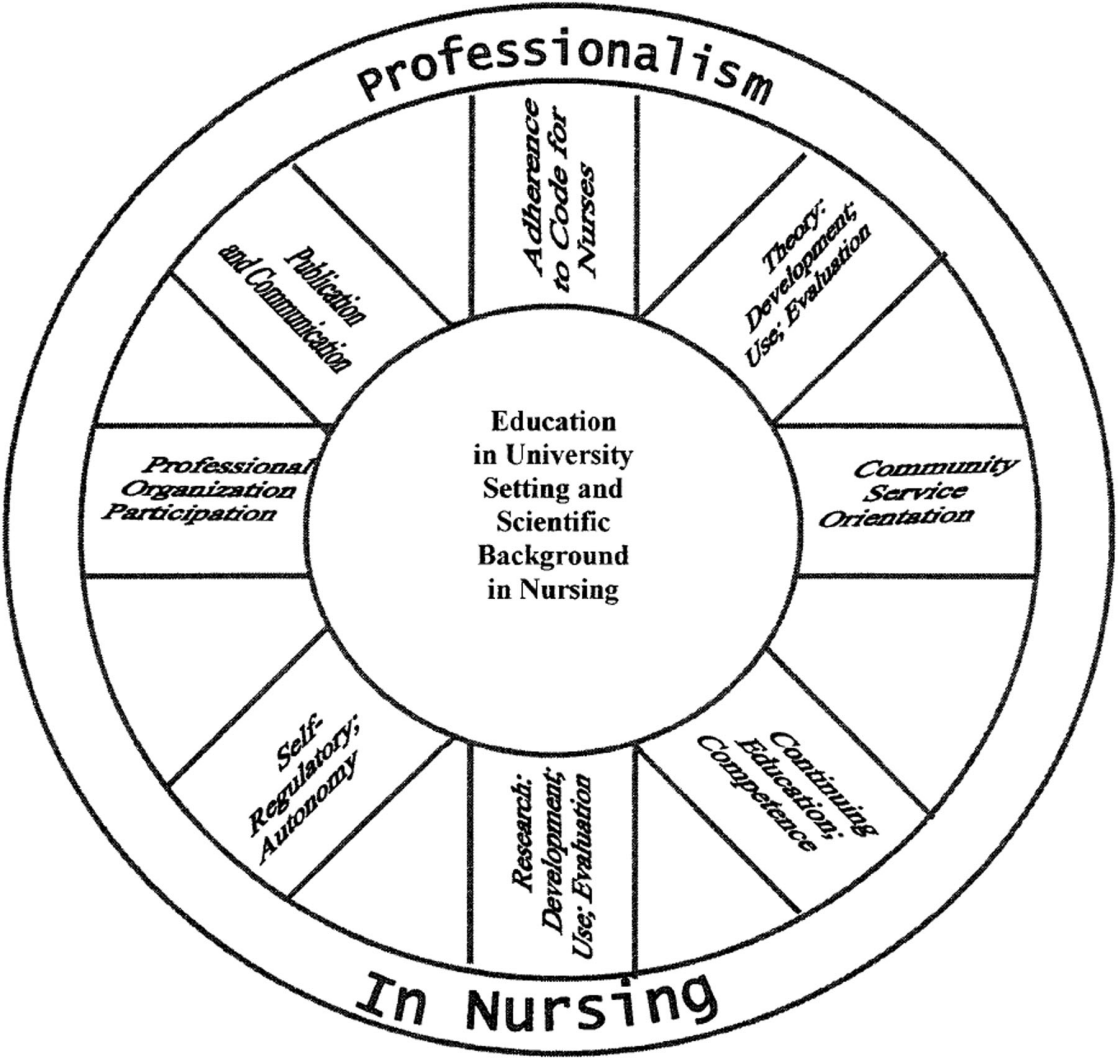
Wheel of Professionalism in Nursing. Reprinted from Adams and Miller (2001), Copyright (2001), with permission from Elsevier.

In relation to professionalism in nursing, Adams and Miller (2001) explored professionalism among nurse practitioners, developing the ‘wheel of professionalism’ (Figure 2). As can be seen in their diagram, ‘education in university setting and scientific background in nursing’ was identified as a nodal point linking diverse concepts such as competence, autonomy, adherence to a code of practice and participation in a professional organisation with ‘Professionalism in Nursing’ as an overarching concept. This demonstrates the transition from ‘elements’ to ‘moments’ through relation to the nodal point; for example, research and theory development are not unique to nursing. When associated with the ‘background in nursing’ nodal point, nursing research and nursing theory are articulated into the discourse of nursing professionalism.

## 8 Difference and Equivalence

Laclau and Mouffe (1985) distinguish between the ‘logic of difference’ and the ‘logic of equivalence’. Equivalence connects signifiers while difference separates them and breaks discursive chains. A nursing example would be the ‘6 C's’ designated by NHS England (2013) as ‘Compassion in Practice’ Values: care, compassion, communication, courage, competence and commitment. Grouping these disparate concepts under an umbrella of ‘values’ connects them through equivalence. However, Laclau and Mouffe (1985, 114) draw attention to ‘the ambiguity penetrating every relation of equivalence: two terms, to be equivalent, must be *different – otherwise there would be a simple identity. On the other hand, the equivalence exists only through the act of subverting the differential character of those terms*’. To continue the previous example, ‘care’ and ‘compassion’ have distinct meanings as singular terms; the equivalence comes through association under ‘Compassion in Practice’ as a nodal point. If a chain of equivalence can be seen emerging from the nodal point of nursing values, the logic of difference here might be competing agendas of task‐focus or efficiency drives, as previously identified within the nursing discourse (Ramsey et al. 2022). As has already been demonstrated, the logic of equivalence has been applied to link diverse concepts within the concept of nursing professionalism.

## 9 Empty Signifiers

Laclau and Mouffe (1985, 98) state that ‘any discourse is constituted as an attempt to dominate the field of discursivity, to arrest the flow of differences, to construct a centre’. They describe unfixed discursive elements as ‘floating signifiers, incapable of being wholly articulated to a discursive chain’ (1985, 99). A floating signifier is invested with different meanings in different discourses; for example, the term ‘body’ might constitute a nodal point within the discourse of nursing, yet acts as a floating signifier in debates between traditional and alternative medicine (Laclau 1990; Jørgensen and Phillips 2011).

While the meaning of floating signifiers shifts across differing perspectives and contexts, as different demands compete over their definition, *empty* signifiers are devoid of specific meaning; their meaning is ‘emptied’ to meet multiple demands. The empty signifier serves to stabilise the discourse in a move towards hegemony (MacKillop 2018; Angouri and Glynos 2009). The concepts of floating and empty signifiers will typically overlap, as Moraes (2014, 30) explains, ‘you will never have a signifier that is so precisely linked to a meaning that the emptiness fully disappears, and you will never have a signifier that is so empty that no reference is included in it’. In terms of chains of equivalence, an empty signifier comes to represent the full chain of signifiers connected to it, a representative of the *equivalence itself* and dividing the discourse into two halves. The empty signifier represents ‘not the other thing’, *not* those signifiers which represent the logic of difference. One example of this is ‘terrorism’ which removes nuance between goodness and evil; ‘when an act is labelled as “terrorist,” its actual traits are blurred, replaced by a mythical set of characteristics’ (Jacobs 2018, 305).

An empty signifier is not attached to a specific signified, instead, it represents a lack or absence. Laclau (2007, 39) describes how an empty signifier demonstrates the limits of signification, ‘the pure negativity of the excluded’. He goes on to note that ‘this relation by which a particular content becomes the signifier of the absent communitarian fullness … is the very condition of hegemony’ (p. 43). In order for differences to be combined into a system, a coherent whole, the signifier is emptied of any independent meaning to provide a name and identity for the system itself, which otherwise would be nameless, ‘a that without a what’ (Linden 2023). Political struggles for hegemony employ empty signifiers such as ‘unity’ or ‘the people’ to denote the lack of a defined common identity (Torfing 1999). This links back to the previous discussion of difficulties defining nursing identity and its subsequent association with professionalism within the nursing literature.

Laclau (2000, 56) explains that ‘the more extended the chain of equivalences that a particular sector comes to represent … the looser will be the links between that name and its original particular meaning, and the more it will approach the status of an empty signifier’. Figure 1, based on a comprehensive analysis of the nursing literature, elaborates several chains of equivalence, each culminating in the nodal point of ‘professionalism’ and also highlights the floating signifiers professional, caring and altruism. For example, the moments evidence‐based practice, lifelong learning, nursing knowledge and innovation are linked by Cao et al. (2023) to the nodal point ‘professional’ in an extended chain of equivalence.

## 10 Professionalism: Antagonism

In the study conducted by Österlind et al. (2011) regarding carers' thoughts and feelings about death, antagonism was identified within the discourse as staff balanced the presence of both life and death. Two discursive positions emerged, one which acknowledged and confronted death, and another which avoided death, holding it at bay. The concept of antagonism is central to discourse theory, being necessary to establish political frontiers and partially fix discursive formations (Howarth 1998; Torfing 1999). As previously discussed, the empty signifier is evidence of ‘antagonism’ as being the point where the discourse splits into difference and equivalence (Jacobs 2018); in this instance, the split is between ‘professional’ and ‘not professional’. Social antagonism occurs when an ‘other’ prevents someone from being true to themselves (Howarth 1998). A person may occupy a subject position articulated by the prevailing discourse of society (worker, parent, woman, etc.), yet experience a ‘lack’ as such positions do not capture their individual identity. This highlights the impossibility of an individual defining their identity through social relations, demonstrating the antagonism at the limits and boundaries of social order (Howarth 1998; MacKillop 2018). Different identities may make competing demands, such as ‘worker’ and ‘parent’; the resulting antagonism threatens the fixity of meaning of the prevailing discourse and therefore its existence (Laclau 1990; Jørgensen and Phillips 2011). This is highly relevant to those of us occupying the subject position ‘nurse’.

In their analysis of nursing treatment plans, Sabab et al. (2020, 10) identified both problem‐focused discourse and care‐focused discourse, though care‐focused discourse was felt to be hegemonic ‘in that it is used most frequently and appears to be the most important and most comprehensive’. They noted that ‘hegemonic care discourse in the nursing records reduces the meaning of the notes to a level of detail that is understandable [only] to those who are familiar with the context. Since the meaning is obscured, the health care and its outcomes are not clearly communicated’. An element of insularity is therefore developed through hegemony of the treatment plan discourse.

## 11 Professionalism: Hegemony

The question remains as to whether ‘professionalism’ as an empty signifier has resulted in a successful hegemonic formulation within the nursing discourse. Links made between professionalism and nursing identity in the literature, as previously highlighted, provide evidence for a hegemonic force. Difficulties with establishing nursing identity amidst the drives of competing ideologies have created the absence which the empty signifier serves to fill. As shown in Figures 1 and 2, ‘professionalism’ as a term does appear to have been emptied of specific meaning to incorporate ‘everything’ about nursing. It has served to organise and stabilise the discursive field around nodal points such as knowledge and altruism, while partially fixing the meaning of floating signifiers such as ‘care’. It is this incorporation of ‘everything’ which determines ‘professionalism’ successful as an empty signifier, highlighting what Laclau describes as ‘the pure negativity of the excluded’; ‘the being or systematicity of the system which is represented through the empty signifiers is … one which is constitutively unreachable’ (Laclau 2007, 39).

There are no set criteria for the definition of a ‘profession’. On a simplistic level, receipt of payment for services differentiates between, for example, an amateur or a professional athlete. In an early attempt to define the concept, Flexner (2001 [1915]) suggested criteria including intellectual endeavour, practical and theoretical skill, autonomy, a dedicated body of knowledge and altruistic motivation. Later, Hall (1968) Professional Inventory Scale measured five ‘professional attitudes’: belief in public service, a sense of calling, autonomy, self‐regulation and use of the professional organisation as a major referent. The majority of these concepts have been incorporated within the chains of equivalence of the nursing discourse, as Cao et al.'s (2023) diagram (Figure 1) demonstrates.

The status of nursing as a profession is still not universally accepted, and the nature of our professionalism has been debated for decades in the nursing literature. In 1998, Rutty (1998, 243) noted that ‘nursing has devoted an extravagant amount of concentration to the subject of professionalism and professionalization’. The complex multidimensionality of the concept has been highlighted, with difficulty in definition linked to differences in meaning across cultures, contexts and time (Sullivan and Thiessen 2015). One definition is that of Miller (1988), who outlined the nine criteria for professionalism in nursing as shown in Figure 2, differing little from those previously elucidated by Hall and Flexner; these included research, use of theory, community service, autonomy, self‐regulation, education, ethics and participation in the professional organisation. According to Miller et al. (1993) the criteria were assembled on the basis that ‘nurses must disclaim the traditional analysis of profession and professionalism by other disciplines as the only method to determine definitions and characteristics of professionalism in nursing’ (p. 290). With evident circularity, the components chosen to represent professionalism were ‘prevalent and traditionally agreed on professionalism characteristics’ based on Nursing Codes, policies and recommendations of professional bodies (Miller et al. 1993, 290).

Defining hegemony, Laclau and Mouffe (1985, xiii) describe how the empty signifier is transformed ‘*in the representation of a universality transcending it (that of the equivalential chain)*. *This* relation, by which a certain particularity assumes the representation of a universality entirely incommensurable with it, is what we call a *hegemonic relation*’. In order to represent the chain of equivalence, the concept of professionalism is ‘transformed’ to encompass all moments within the chain. The concept analysis undertaken by Cao et al. (2023) identified many moments in the chain of equivalence, including clinical knowledge, continuing professional development, evidence‐based practice, striving for excellence, innovation, dedication and public service. ‘Care’ was described as the core attribute of professionalism, a nodal point for concepts such as concern, kindness and empathy, creation of a healing environment and demonstrating altruism. Many of these attributes would seem to be more fitting to a definition of ‘nursing’ itself, which brings to light the effectiveness of ‘professionalism’ as an empty signifier within the discourse; the ‘emptiness’ is a lack of an alternative coherent identity for nursing, other than ‘not un‐professional’. As Torfing (1999, 120) describes, ‘the hegemonic force, which is responsible for the negation of individual or collective identity, will tend to construct the excluded identity as one of a series of threatening obstacles to the full realization of chosen meanings and options’.

To determine whether the particularity ‘professionalism’ has successfully assumed ‘the representation of a universality’, it would be useful to briefly consider the development of nursing as a profession. For many years, other scientists considered nursing a semi‐professional career (Adams and Miller 2001). Davies (1995, 134) describes the ‘quest for full professional status’ undertaken by nurse leaders of the late 19th Century to the late 20th Century. Until the 1970s, nursing was typically considered to be ‘women's work’, which of itself was viewed as a barrier to professionalism. Davies (1995) described nursing as ‘a much‐conflicted metaphor in our culture, reflecting all the ambivalences we give to the meaning of womanhood’ (p. 179). At this time, there was a limited theory base, particularly in relation to the ‘science’ of nursing, and educational requirements varied considerably (Adams and Miller 2001). The body of knowledge necessary for professionalism began to emerge through development of nursing models and theories in America in the 1970s (Traynor 1996). The 1972 Briggs report declared that ‘nursing must become an evidence‐based profession’ and through the 1980s nursing focused increasingly on science and research, leading Raatikainen (1989) to ask whether such shifts would alter the fundamental nature of the profession. It was felt that research could improve the cost‐effectiveness of nursing (Perry and Jolley 1991) with emphasis on scientific and technical aspects of the role viewed as necessary to increase prestige and status (Ford and Walsh 1994). van der Cingel and Brouwer (2021, 3) describe a dualistic split between ‘thinking’ and ‘doing’ related to autonomy in day‐to‐day work; while nursing is typically viewed as ‘a practical, doing, down to earth job’, there is a vast nursing knowledge base to underpin this. It is apparent that nursing identity has been significantly impacted by the effects of gender inequality and patriarchal power and swayed by competing ideologies, including regulatory power, with consequent emphasis on professionalism.

According to Laclau and Mouffe (1985, 112), antagonism is ‘the “experience” of the limit of the social … *antagonisms constitute the limits of society, the latter's impossibility of fully constituting itself*’. Nursing has always struggled to be ‘fully constituted’; with the antagonistic frontier in this instance serving to delineate the ‘women's work’ of caring and nurturance from the cost‐effectiveness of evidence‐based practice. The success of ‘professionalism’, therefore, is in integrating both scientific and artistic elements of nursing work, both caring and cost‐effectiveness, into a single hegemonic discourse. Laclau (2007, 43) further notes that ‘a class or group is considered to be hegemonic when it … presents itself as realizing the broader aims either of emancipating or ensuring order for wider masses of the population’. A religious fervour for evidence‐based practice and slavish adherence to guidelines could certainly be viewed in terms of ‘ensuring order’. Given that there is now considerably less debate both outside and within nursing regarding whether it is a ‘profession’, it seems that the empty signifier ‘professionalism’ has made significant strides towards hegemony. It also serves to highlight the lack of an alternative, coherent identity for nursing, with the empty signifier defining us merely as ‘not unprofessional’. However, descriptions of ‘unprofessional behaviour’ within the nursing literature typically refer to failure to meet *professional standards*, as set out in *professional guidelines* and codes of conduct (Papinaho et al. 2022). A brief survey of the literature identifies multiple practices deemed ‘unprofessional’ within nursing, including bullying and incivility (Livshiz‐Riven et al. 2023), failure to respect patient rights, including dignity and threatening the safety of patients (Papinaho et al. 2022).

## 12 Impact for Nursing

Using Laclau and Mouffe's (1985) discourse theory, ‘professionalism’ has been demonstrated to provide structure in the construction of nursing identity. Elements within the discourse have meaning specific to nursing, such as ‘observation’ or ‘turn’; such meanings are fixed through association to nodal points such as ‘hospital ward’ or ‘pressure relief’. Nodal points can also create chains of equivalence, for example, linking the elements compassion and care as nursing values. Chains of equivalence relating to competence, values and autonomy, among others, are linked by the nodal point and empty signifier of professionalism; it is the equivalence itself which professionalism represents. Multiple key components of nursing identity have been subsumed within the concept of professionalism, such as accountability and compassion, terms which themselves are discursively constructed and might benefit from further exploration. It therefore seems worthwhile to address the concept of professionalism as an empty signifier, given its prominence within the discourse. As demonstrated, application of discourse theory within nursing has the potential to create a rudimentary shift in thinking about nursing identity and is likely applicable to multiple other aspects of nursing discourse. Hegemonic movements occur insidiously, often outside conscious awareness; by unpicking the elements of the discourse and considering the sociopolitical trends underpinning the drive towards hegemony, discourse analysis takes on greater depth.

While Laclau and Mouffe's work is now 40 years old, it retains a timeless appeal; our use of language, the discursive structure of society, and the pull of competing ideologies are as relevant today as they ever were. Laclau and Mouffe (1985) developed their theory at a time when activist organisations were beginning their drive towards inclusivity, a movement which remains highly relevant. A major focus of radical democracy was to highlight and challenge oppressive power relations; this seems highly applicable to current nursing practice. For example, Dillard‐Wright and Jenkins (2024, 25) describe nursing as ‘a total institution’ within which nurses are subject to oppression and control ‘according to the metrics of healthcare productivity, quality, and compliance’. Nursing education, along with professional bodies and guidelines, serves to maintain this status quo, as ‘control over nurse identity and the power to define who nurses are and what nurses do are central to the intersection of power, agency, governance, and oppression’. Given the increased number of healthcare worker strikes worldwide, among other forms of protest, it would be interesting to explore future potential for a shift towards an *emancipatory* discourse for nursing and the evolution of nursing identity, as elaborated by Dillard‐Wright and Jenkins (2024).

Rutty (1998) discussed the importance of nursing philosophy in achieving professional status for nursing. This links to the dualism between ‘doing’ and ‘thinking’ identified by van der Cingel and Brouwer (2021). They go on to describe the image of nursing as ‘female’ as persisting in the public imaginary, with media representation of nurses rife with inaccurate stereotyping. Given that discourse theory deals with the ‘logic of the social’, it seems ideally situated to explore the complex societal and cultural dynamics behind the persistence of such an archaic view, along with the role of ideology in the development of nursing identity. The position of nursing within wider discourse could therefore be considered, along with exploration of further aspects of the nursing discourse itself.

## 13 Conclusion

Through the identification of the empty signifier ‘professionalism’ and the exploration of the ensuing hegemonic formation of ‘professional’ nursing identity, the work of Laclau and Mouffe has been demonstrated to have significant relevance to nursing. If the hegemonic discourse of nursing identity has been constructed around an empty signifier, it seems highly relevant to draw attention to this, the underlying mechanism by which this has occurred and the potentiality for an emancipatory discourse. As outlined above, there are likely to be many further instances where interrogation of the nursing discourse through the lens of Laclau and Mouffe's Discourse Theory may prove fruitful.

## Conflicts of Interest

The author declares no conflicts of interest.

## Data Availability

The author has nothing to report.

## References

[nup70034-bib-0001] Adams, D. , and B. K. Miller . 2001. “Professionalism in Nursing Behaviors of Nurse Practitioners.” Journal of Professional Nursing 17, no. 4: 203–210. 11464342 10.1053/jpnu.2001.25913

[nup70034-bib-0002] Althusser, L. 1969. For Marx. Penguin.

[nup70034-bib-0003] Althusser, L. 1971. “Ideology and Ideological State Apparatuses.” In Lenin, Philosophy, and Other Essays, edited by L. Althusser , 128–186. New Left Books.

[nup70034-bib-0004] Althusser, L. 1990. Philosophy and the Spontaneous Philosophy of the Scientists and Other Essays. Verso Books.

[nup70034-bib-0005] Angouri, J. , and J. Glynos . 2009. Managing Cultural Difference and Struggle in the Context of the Multinational Corporate Workplace: Solution or Symptom? (Working Paper). University of Essex.

[nup70034-bib-0006] Aranda, K. 2019. “The Political Matters: Exploring Material Feminist Theories for Understanding the Political in Health, Inequalities and Nursing.” Nursing Philosophy 20, no. 4: e12278. 31364816 10.1111/nup.12278

[nup70034-bib-0007] Bass, J. 2005. “Measures of Democracy.” In Encyclopaedia of Social Measurement, edited by K. Kempf‐Leonard , 637–643. Elsevier.

[nup70034-bib-0008] Biglieri, P. , and G. Perelló . 2011. “The Names of the Real in Laclau's Theory: Antagonism, Dislocation, and Heterogeneity.” Filozofski Vestnik 32, no. 2: 47–64.

[nup70034-bib-0009] Cao, H. , Y. Song , Y. Wu , et al. 2023. “What Is Nursing Professionalism? A Concept Analysis.” BMC Nursing 22, no. 1: 34. 36747180 10.1186/s12912-022-01161-0 PMC9902819

[nup70034-bib-0011] Cloyes, K. G. 2002. “Agonizing Care: Care Ethics, Agonistic Feminism and a Political Theory of Care.” Nursing Inquiry 9, no. 3: 203–214. 12199885 10.1046/j.1440-1800.2002.00147.x

[nup70034-bib-0013] Dahlberg, L. 2012. “Radical Democracy.” In The Edinburgh Companion to the History of Democracy (Chapter 41), edited by B. Isakhan and S. Stockwell , 491–501. Edinburgh University Press.

[nup70034-bib-0014] Davies, C. 1995. Gender and the Professional Predicament in Nursing. Open University Press.

[nup70034-bib-0015] Dillard‐Wright, J. , and D. Jenkins . 2024. “Dangerous and Unprofessional Content: Anarchist Dreams for Alternate Nursing Futures.” Philosophies 9, no. 1: 25.

[nup70034-bib-0016] Felder, M. , S. Kuijper , P. Lalleman , R. Bal , and I. Wallenburg . 2022. “The Rise of the Partisan Nurse and the Challenge of Moving Beyond an Impasse in the (Re) Organization of Dutch Nursing Work.” Journal of Professions and Organization 9, no. 1: 20–37.

[nup70034-bib-0017] Flexner, A. 2001 [1915]. “Is Social Work a Profession?.” Research on Social Work Practice 11, no. 2: 152–165.

[nup70034-bib-0018] Ford, P. , and M. Walsh . 1994. New Rituals for Old: Nursing Through the Looking Glass. Butterworth Heinemann.

[nup70034-bib-0019] Forgacs, D. , ed. 2000. The Gramsci Reader: Selected Writings, 1916–1935. New York University Press.

[nup70034-bib-0024] Ghadirian, F. , M. Salsali , and M. A. Cheraghi . 2014. “Nursing Professionalism: An Evolutionary Concept Analysis.” Iranian Journal of Nursing and Midwifery Research 19, no. 1: 1–10. 24554953 PMC3917177

[nup70034-bib-0025] Hall, R. H. 1968. “Professionalization and Bureaucratization.” American Sociological Review 33: 92–104.

[nup70034-bib-0027] Howarth, D. 1995. “Discourse Theory.” In Theory and Methods in Political Science, edited by D. Marsh and G. Stoker , 115–136. Macmillan.

[nup70034-bib-0028] Howarth, D. 1998. “Discourse Theory and Political Analysis.” In Research Strategies in the Social Sciences: A Guide to New Approaches, edited by E. Scarbrough and E. Tanenbaum , 268–293. Oxford University Press.

[nup70034-bib-0029] Hunt, G. , and P. Wainwright . 1994. Expanding the Role of the Nurse. Blackwell Science.

[nup70034-bib-0030] Jacobs, T. 2018. “The Dislocated Universe of Laclau and Mouffe: An Introduction to Post‐Structuralist Discourse Theory.” Critical Review 30, no. 3–4: 294–315.

[nup70034-bib-0031] Jørgensen, M. , and L. Phillips . 2011. “Discourse Analysis as Theory and Method.” In Sage Research Methods. 10.4135/9781849208871.

[nup70034-bib-0034] Laclau, E. 1990. New Reflections on the Revolution of Our Time. Verso.

[nup70034-bib-0035] Laclau, E. 1993. “Discourse.” In A Companion to Contemporary Political Philosophy, edited by R. Goodin and P. Pettit , 431–437. Blackwell.

[nup70034-bib-0036] Laclau, E. 1998. “Gramsci.” In A Companion to Continental Philosophy, edited by S. Critchley and W. Schroeder , 461–468. Blackwell.

[nup70034-bib-0037] Laclau, E. 2000. “Identity and Hegemony: The Role of Universality in the Constitution of Political Logics.” In Contingency, Hegemony, Universality: Contemporary Dialogues on the Left, edited by J. Butler , E. Laclau and S. Žižek , 44–89. Verso.

[nup70034-bib-0040] Laclau, E. 2007. “Why Do Empty Signifiers Matter to Politics?” In Emancipations, 36–46. Verso.

[nup70034-bib-0041] Laclau, E. , and C. Mouffe . 1985. Hegemony and Socialist Strategy: Towards a Radical Democratic Politics. Verso.

[nup70034-bib-0043] Leopold, D. 2013. “Marxism and Ideology: From Marx to Althusser.” In The Oxford Handbook of Political Ideologies, edited by M. Freeden , L. Sargent and M. Stears , 20–37. Oxford University Press.

[nup70034-bib-0044] Linden, F. R. 2023. “How ‘Empty’ Is the Signifier ‘The People’? Impasses of the Poststructuralist Approach in Ernesto Laclau's Political Ontology.” Journal of Political Ideologies 30, no. 2 (July): 327–351.

[nup70034-bib-0045] Livshiz‐Riven, I. , N. Hurvitz , K. Grinberg , et al. 2023. “Nursing Students' Experiences of Unprofessional Behaviours and Associations With Guideline Compliance: A Multicenter Survey.” Nurse Education in Practice 71: 103739. 37536180 10.1016/j.nepr.2023.103739

[nup70034-bib-0046] MacKillop, E. 2018. “How Do Empty Signifiers Lose Credibility? The Case of Commissioning in English Local Government.” Critical Policy Studies 12, no. 2: 187–208. 10.1080/19460171.2016.1236740.

[nup70034-bib-0047] Matthews, J. , A. Bialocerkowski , and M. Molineux . 2019. “Professional Identity Measures for Student Health Professionals – A Systematic Review of Psychometric Properties.” BMC Medical Education 19: 308. 10.1186/s12909-019-1660-5. 31409410 PMC6693256

[nup70034-bib-0048] Miller, B. 1988. “A Model for Professionalism in Nursing.” Today's OR Nurse 10, no. 9: 18–23. 3176136

[nup70034-bib-0080] Miller, B. K. , D. Adams , and L. Beck . 1993. “A Behavioral Inventory for Professionalism in Nursing.” Journal of Professional Nursing 9, no. 5: 290–295. 8294646 10.1016/8755-7223(93)90055-h

[nup70034-bib-0050] Moraes, S. E. 2014. “Global Citizenship as a Floating Signifier.” International Journal of Development Education and Global Learning 6, no. 2: 27–42.

[nup70034-bib-0051] NHS England . 2013. Compassion in Practice – Implementation. NHS England. https://www.england.nhs.uk/2013/04/cip-implementation/.

[nup70034-bib-0053] Österlind, J. , G. Hansebo , J. Andersson , B. M. Ternestedt , and I. Hellström . 2011. “A Discourse of Silence: Professional Carers Reasoning About Death and Dying in Nursing Homes.” Ageing & Society 31, no. 4: 529–544.

[nup70034-bib-0054] Oute, J. 2018. “‘It Is a Bit Like Being a Parent’: A Discourse Analysis of How Nursing Identity Can Contextualize Patient Involvement in Danish Psychiatry.” Nordic Journal of Nursing Research 38, no. 1: 1–10.

[nup70034-bib-0055] Papinaho, O. , A. Häggman‐Laitila , and M. Kangasniemi . 2022. “Unprofessional Conduct by Nurses: A Document Analysis of Disciplinary Decisions.” Nursing Ethics 29, no. 1: 131–144. 34583555 10.1177/09697330211015289 PMC8866744

[nup70034-bib-0056] Perry, A. , and M. Jolley . 1991. Nursing: A Knowledge Base for Practice. Edward Arnold.

[nup70034-bib-0057] Philippa, R. , H. Ann , M. Jacqueline , and A. Nicola . 2021. “Professional Identity in Nursing: A Mixed Method Research Study.” Nurse Education in Practice 52: 103039. 33823376 10.1016/j.nepr.2021.103039

[nup70034-bib-0058] Phillips, A. 2010. “What's Wrong With Essentialism?.” Distinktion: Journal of Social Theory 11, no. 1: 47–60.

[nup70034-bib-0059] Raatikainen, R. 1989. “Values and Ethical Principles in Nursing.” Journal of Advanced Nursing 14: 92–96. 2649527 10.1111/j.1365-2648.1989.tb00905.x

[nup70034-bib-0061] Ramsey, S. M. , J. Brooks , M. Briggs , and C. E. Hallett . 2022. “Corporatising Compassion? A Contemporary History Study of English NHS Trusts' Nursing Strategy Documents.” Nursing Inquiry 29, no. 4: e12486. 35266239 10.1111/nin.12486

[nup70034-bib-0062] Roberts, P. , and P. Sutch . 2012. An Introduction to Political Thought (2nd ed.). Edinburgh University Press.

[nup70034-bib-0064] Rutty, J. E. 1998. “The Nature of Philosophy of Science, Theory and Knowledge Relating to Nursing and Professionalism.” Journal of Advanced Nursing 28, no. 2: 243–250. 9725719 10.1046/j.1365-2648.1998.00795.x

[nup70034-bib-0065] Sabab, N. , A. Moen , and H. Bondevik . 2020. “Language Use and Patterns in Nursing Records. A Discourse Analytical Approach.” Sykepleien Forskning 15: e‐82323.

[nup70034-bib-0066] Saussure, F. d. 1959. Course in General Linguistics. Translated by C. Bailey and A. Sechehaye. McGraw‐Hill.

[nup70034-bib-0067] Smith, A. 1998. Laclau and Mouffe: The Radical Democratic Imaginary. Routledge.

[nup70034-bib-0068] Sullivan, T. M. , and A. K. Thiessen . 2015. “Occupational Therapy Students' Perspectives of Professionalism: An Exploratory Study.” Open Journal of Occupational Therapy 3, no. 9: 4.

[nup70034-bib-0069] Torfing, J. 1999. New Theories of Discourse: Laclau, Mouffe and Žižek. Wiley‐Blackwell.

[nup70034-bib-0070] Traynor, M. 1996. “Looking at Discourse in a Literature Review of Nursing Texts.” Journal of Advanced Nursing 23, no. 6: 1155–1161. 8796463 10.1046/j.1365-2648.1996.12612.x

[nup70034-bib-0071] Traynor, M. 2019. “Autonomy and Caring: Towards a Marxist Understanding of Nursing Work.” Nursing Philosophy 20, no. 4: e12262. 31197925 10.1111/nup.12262

[nup70034-bib-0074] van der Cingel, M. , and J. Brouwer . 2021. “What Makes a Nurse Today? A Debate on the Nursing Professional Identity and Its Need for Change.” Nursing Philosophy 22, no. 2: e12343. 33450124 10.1111/nup.12343

[nup70034-bib-0075] Williams, B. 2021. “The ‘New Right’ and Its Legacy for British Conservatism.” Journal of Political Ideologies 29, no. 1: 121–144.

